# Widespread retention of ohnologs in key developmental gene families following whole-genome duplication in arachnopulmonates

**DOI:** 10.1093/g3journal/jkab299

**Published:** 2021-09-06

**Authors:** Amber Harper, Luis Baudouin Gonzalez, Anna Schönauer, Ralf Janssen, Michael Seiter, Michaela Holzem, Saad Arif, Alistair P McGregor, Lauren Sumner-Rooney

**Affiliations:** 1 Department of Biological and Medical Sciences, Faculty of Health and Life Sciences, Oxford Brookes University, Oxford OX3 0BP, UK; 2 Department of Earth Sciences, Uppsala University, Geocentrum, 752 36 Uppsala, Sweden; 3 Department of Evolutionary Biology, Unit Integrative Zoology, University of Vienna, 1090 Vienna, Austria; 4 Division of Signalling and Functional Genomics, German Cancer Research Centre (DKFZ), Heidelberg, Germany and Department of Cell and Molecular Biology, Medical Faculty Mannheim, Heidelberg University, 69120 Heidelberg, Germany; 5 Centre for Functional Genomics, Oxford Brookes University, Oxford OX3 0BP, UK; 6 Oxford University Museum of Natural History, University of Oxford, Oxford OX1 3PW, UK

**Keywords:** arachnids, developmental toolkit, gene duplication, transcriptomes, whole-genome duplication

## Abstract

Whole-genome duplications (WGDs) have occurred multiple times during animal evolution, including in lineages leading to vertebrates, teleosts, horseshoe crabs, and arachnopulmonates. These dramatic events initially produce a wealth of new genetic material, generally followed by extensive gene loss. It appears, however, that developmental genes such as homeobox genes, signaling pathway components and microRNAs are frequently retained as duplicates (so-called ohnologs) following WGD. These not only provide the best evidence for WGD, but an opportunity to study its evolutionary consequences. Although these genes are well studied in the context of vertebrate WGD, similar comparisons across the extant arachnopulmonate orders are patchy. We sequenced embryonic transcriptomes from two spider species and two amblypygid species and surveyed three important gene families, *Hox*, *Wnt*, and *frizzled*, across these and 12 existing transcriptomic and genomic resources for chelicerates. We report extensive retention of putative ohnologs, further supporting the ancestral arachnopulmonate WGD. We also found evidence of consistent evolutionary trajectories in *Hox* and *Wnt* gene repertoires across three of the six arachnopulmonate orders, with interorder variation in the retention of specific paralogs. We identified variation between major clades in spiders and are better able to reconstruct the chronology of gene duplications and losses in spiders, amblypygids, and scorpions. These insights shed light on the evolution of the developmental toolkit in arachnopulmonates, highlight the importance of the comparative approach within lineages, and provide substantial new transcriptomic data for future study.

## Introduction

The duplication of genetic material is an important contributor to the evolution of morphological and physiological innovations (Ohno 1970; Zhang 2003). The most dramatic example of this is whole-genome duplication (WGD), when gene copy numbers are doubled and retained paralogs (ohnologs) can then share ancestral functions (subfunctionalization) and/or evolve new roles (neofunctionalization; Ohno 1970; Force *et al.* 1999; Lynch and Conery 2000). The occurrence of two rounds (2R) of WGD in the early evolution of vertebrates has long been associated with their taxonomic and morphological diversity (*e.g.*, Ohno 1970; Holland *et al.* 1994; Dehal and Boore 2005; Holland 2013a), and a subsequent 3R in teleosts is frequently linked to their success as the most diverse vertebrate group (*e.g.*, Meyer and Schartl 1999; Glasauer and Neuhauss 2014). However, this remains controversial and difficult to test (Donoghue and Purnell 2005) and in several animal lineages, there is no clear association between WGD and diversification (Mark Welch *et al.* 2008; Flot *et al.* 2013; Havlak *et al.* 2014; Kenny *et al.* 2016; Nong *et al.* 2020). Along with vertebrates, chelicerates also appear to be hotspots of WGD, with up to three rounds reported in horseshoe crabs (Havlak *et al.* 2014; Kenny *et al.* 2016; Nong *et al.* 2020; Shingate *et al.* 2020), and potentially two further rounds within the spider clade Synspermiata (Král *et al.* 2019). Chelicerates demonstrate a highly variable body plan, occupy a wide range of habitats and ecological niches, and have evolved a variety of biologically important innovations such as venoms and silks (Schwager *et al.* 2015). They therefore offer an excellent opportunity for comparison with vertebrates concerning the implications of WGD for morphological and taxonomic diversity, and genome evolution in its wake.

The house spider *Parasteatoda tepidariorum* has emerged as a model species to study the impacts of WGD on arachnid evolution and development. Genomic and functional developmental studies have found retained ohnologs of many important genes, with evidence for neo- and subfunctionalization compared to single-copy orthologs in arachnids lacking WGD (Janssen *et al.* 2015; Leite *et al.* 2016; Turetzek *et al.* 2016, 2017; Schwager *et al.* 2017; Leite *et al.* 2018; Baudouin-Gonzalez *et al.* 2021). Work on the scorpions *Centruroides sculpturatus* and *Mesobuthus martensii* has consistently complemented findings in *P. tepidariorum*, with genomic studies recovering many ohnologs retained in common with spiders (Sharma *et al.* 2014b; Di *et al.* 2015; Sharma *et al.* 2015; Schwager *et al.* 2017; Leite *et al.* 2018). In the past few years, several additional spider genomes have become available, providing an opportunity to get a more detailed view of genome evolution following WGD. Although synteny analysis remains the gold standard for the identification of ohnologs, the required chromosome-level genomic assemblies remain relatively scarce. Work on the *P. tepidariorum, C. sculpturatus*, and *M. martensii* genomes has been complemented by targeted studies of individual gene families and transcriptomic surveys (Schwager *et al.* 2007; Sharma *et al.* 2012; Janssen *et al.* 2015; Leite *et al.* 2018; Gainett and Sharma 2020; Baudouin-Gonzalez *et al.* 2021). Combined with phylogenetic analyses, the identification of duplications can provide support for WGD events and their timing in arachnid evolution. Although transcriptomes can yield variant sequences of individual genes, from different alleles or individuals in mixed samples, these are generally straight-forward to filter out from truly duplicated loci owing to substantial sequence divergence in the latter. They also offer the double-edged sword of capturing gene expression, rather than presence in the genome; pseudogenized or silenced duplicates are not detected, but neither are functional genes if they are not expressed at the sampled timepoint or tissue. Such studies have produced strong additional evidence for an ancestral WGD, with patterns of duplication coinciding with our expectations for arachnopulmonate ohnologs (Clarke *et al.* 2014; 2015; Sharma *et al.* 2015; Turetzek *et al.* 2017; Bonatto Paese *et al.* 2018; Leite *et al.* 2018; Gainett *et al.* 2020; Gainett and Sharma 2020; Baudouin-Gonzalez *et al.* 2021).

Comparison of WGD events among arachnopulmonates, horseshoe crabs, and vertebrates indicates that despite extensive gene loss following duplication events, certain gene families are commonly retained following duplication (Holland *et al.* 1994; Schwager *et al.* 2007; Kuraku and Meyer 2009; Di *et al.* 2015; Sharma *et al.* 2015; Kenny *et al.* 2016; Leite *et al.* 2016, 2018; Schwager *et al.* 2017). These typically include genes from the conserved developmental “toolkit” of transcription factors (TFs), cell signaling ligands and receptors, and microRNAs (Erwin 2009). Among these, several have stood out as focal points in the study of gene and genome duplications. The *Hox* group of homeobox genes regulate the identity of the body plan along the antero-posterior axis of all bilaterian animals (McGinnis and Krumlauf 1992; Abzhanov *et al.* 1999; Carroll *et al.* 2005; Pearson *et al.* 2005; Hueber and Lohmann 2008; Holland 2013b). Four clusters of these key developmental genes were partially retained after 1R and 2R in vertebrates (Holland *et al.* 1994; Meyer and Schartl 1999; Kuraku and Meyer 2009; Pascual-Anaya *et al.* 2013), and the arachnopulmonate WGD is evident in the almost universal retention of *Hox* gene duplicates in sequenced genomes, with two ohnologs of all 10 arthropod *Hox* genes in the scorpion *M. martensii* (Di *et al.* 2015; Leite *et al.* 2018), all except *Hox3* being represented by two copies in *C. sculpturatus* (Leite *et al.* 2018), and all except *fushi tarazu* (*ftz*) in *P. tepidariorum* (Schwager *et al.* 2017). Systematic studies of *Hox* gene expression patterns in the latter demonstrated that all nine pairs of *Hox* paralogs exhibit signs of sub- or neofunctionalization (Schwager *et al.* 2017). This high level of retention and expression divergence lends strong support to the importance of *Hox* gene duplication in the evolution of the arachnopulmonate body plan, and further consolidates the position of this gene family as a key indicator of WGD. Additionally, arachnids lacking WGD, such as ticks, mites, and harvestmen, exhibit single copies of the *Hox* genes, with no evidence for duplication via other mechanisms (Grbić *et al.* 2011; Pace *et al.* 2016; Leite *et al.* 2018; Gainett *et al.* 2021).

In addition to TFs, the ligands and receptors of some signaling pathways of the developmental toolkit (*e.g.*, Hedgehog, Wnt, TGF-ß, NHR) also demonstrate higher copy numbers in vertebrates and other groups subject to WGD, including arachnopulmonates (Holland *et al.* 1994; Meyer and Schartl 1999; Shimeld 1999; Pires-daSilva and Sommer 2003; Cho *et al.* 2010; Janssen *et al.* 2010; Hogvall *et al.* 2014; Janssen *et al.* 2015). The Wnt signaling pathway plays many important roles during animal development, including segmentation and patterning of the nervous system, eyes, and gut (Erwin 2009; Murat *et al.* 2010). In the canonical pathway, Wnt ligands bind to transmembrane receptors, such as Frizzled, to trigger translocation of ß-catenin to the nucleus and mediate regulation of gene expression (Cadigan and Nusse 1997; Hamilton *et al.* 2001; Logan and Nusse 2004; van Amerongen and Nusse 2009). There are 13 subfamilies of *Wnt* genes found in bilaterians, as well as multiple receptor families and downstream components. In contrast to the extensive retention of *Hox* ohnologs following WGD, *Wnt* duplicates in *P. tepidariorum* appear to be restricted to *Wnt7* and *Wnt11*, with the remaining eight subfamilies represented by single genes (Janssen *et al.* 2010). However, these are the only reported *Wnt* gene duplications in arthropods despite several recent surveys (Bolognesi *et al.* 2008; Murat *et al.* 2010; Hayden and Arthur 2013; Meng *et al.* 2013; Chipman *et al.* 2014; Hogvall *et al.* 2014; Janssen and Posnien 2014; Kao *et al.* 2016; Holzem *et al.* 2019), and beyond *P. tepidariorum* no other arachnopulmonates have been systematically searched.

Several *Wnt* families have also been retained after the 1R and 2R events in vertebrates, for example there are two copies each of *Wnt2*, *Wnt3*, *Wnt5*, *Wnt7*, *Wnt8*, *Wnt9*, and *Wnt10* in humans (Miller 2001; Janssen *et al.* 2010). However, no subfamilies are represented by three or four copies in humans and so there is some consistency with arachnopulmonates in that the *Wnts* may be more conservative markers of WGD, to be used in combination with *Hox* and other homeobox genes.

Similarly, duplications within the four *frizzled* gene subfamilies appear to be restricted to arachnopulmonates among arthropods, wherein only *fz4* is duplicated in both *P. tepidariorum* and *M. martensii* (Janssen *et al.* 2015).

The extensive and consistent retention of key developmental genes, like *Hox* genes apparent in *P. tepidariorum* and *C. sculpturatus*, and *Wnt* genes in *P. tepidariorum*, strongly support the occurrence of an ancestral WGD in arachnopulmonates. However, data are only available for a handful of species so far, resulting in very patchy taxonomic sampling. For example, only *P. tepidariorum*, *Pholcus phalangioides* and recently, *Aphonopelma hentzi*, have been comprehensively surveyed for homeobox genes among spiders (Leite *et al.* 2018; Ontano *et al.* 2021), omitting the large and derived retrolateral tibial apophysis (RTA) clade, which includes jumping spiders, crab spiders, and other free hunters, and the systematic identification of *Wnt* genes has been restricted to only *P. tepidariorum*. Spiders and scorpions are by far the most speciose of the arachnopulmonates, and there may be additional diversity in their repertoires of these important developmental gene families of which we are not yet aware.

In addition, and perhaps more urgently, only two of the six arachnopulmonate lineages have dominated the field thus far; sufficient genomic information for comparison is lacking beyond spiders and scorpions. Also represented in Arachnopulmonata are the amblypygids (whip spiders), relatively understudied and enigmatic animals comprising around 190 extant species. They exhibit highly derived pedipalp morphology, which are adapted to form raptorial appendages, and of the first pair of walking legs, which are antenniform and can comprise more than 100 segments (Weygoldt 2009). Despite the scarcity of transcriptomic or genomic data for amblypygids [see Gainett *et al.* (2020) for recent advances; Gainett and Sharma (2020)], their widely accepted position within Arachnopulmonata implies that they were also subject to an ancestral WGD. A recent survey of the *Phrynus marginemaculatus* transcriptome supported this in the recovery of multiple duplicate *Hox* and leg gap genes (Gainett and Sharma 2020). Particularly given the derived nature of their appendages, this group could shed substantial light on genomic and morphological evolution following WGD. The position of pseudoscorpions was also recently resolved within Arachnopulmonata, complemented by widespread homeobox gene duplication (Ontano *et al.* 2021).

To better understand the genomic consequences of WGD in a greater diversity of arachnopulmonate lineages, we sequenced *de novo* embryonic transcriptomes from two spiders belonging to the derived RTA clade and two amblypygids. We surveyed *Hox*, *Wnt*, and *frizzled* genes in these species and existing genomic and transcriptomic resources for comparison with other arachnids, both with and without an ancestral WGD, improving sampling at both the order and suborder levels.

## Materials and methods

### Embryo collection and fixation

Embryos of mixed ages were collected from captive females of the amblypygids *Charinus acosta* (Charinidae; parthenogenetic, collected at 1 day, 1 month, and 2 months after laying; equivalent to approximately 1%, 30%, and 60% of development) and *Euphrynichus bacillifer* (Neoamblypygi: Phrynichidae; mated, collected at approximately 30% of development), the wolf spider *Pardosa amentata* (collected in Oxford, UK, equivalent to stages 11/12 in *P. tepidariorum*) and mixed-stage embryos of the jumping spider *Marpissa muscosa* (kindly provided by Philip Steinhoff and Gabriele Uhl, University of Greifswald) and stored in RNAlater. *Phalangium opilio* were collected in Uppsala, Sweden, and developmental series of embryos ranging from egg deposition to the end of embryogenesis were collected for sequencing.

### Transcriptomics

We extracted total RNA from embryos, pooled by species, of *C. acosta*, *E. bacillifer*, *P. amentata*, and *M. muscosa* using QIAzol lysis reagent and following the manufacturer’s instructions (Qiagen). Libraries were prepared using a TruSeq RNA kit (including polyA selection) and sequenced on the NovaSeq platform (100 bp PE, Edinburgh Genomics). Following quality assessment using FastQC v0.11.9 (Andrews 2010), erroneous k-mers were removed (rCorrector, default settings; Song and Florea 2015), and unfixable read pairs (from low-expression homolog pairs or containing too many errors) were discarded using a custom Python script (available at https://github.com/harvardinformatics/TranscriptomeAssemblyTools/blob/master/FilterUncorrectabledPEfastq.py courtesy of Adam Freeman). Adapter sequences were identified and removed and low-quality ends (phred score cutoff = 5) trimmed using TrimGalore! v0.6.5 (available at https://github.com/FelixKrueger/TrimGalore). *De novo* transcriptome assembly was performed using only properly paired reads with Trinity v2.10.0 (Haas *et al.* 2013) using default settings. Transcriptome completeness was assessed on the longest isoform per gene using BUSCO v4.0.2 (Seppey *et al.* 2019), the arachnid database (arachnida_odb10 created on 2019-11-20; 10 species, 2934 BUSCOs), and the arthropod database (arthropoda_odb10 created on 2019-11-20; 90 species, 1013 BUSCOs). Reads and assembled transcriptomes are available on SRA and TSA, respectively, under BioProject PRJNA707377.

### Identification of gene candidates

To identify *Hox*, *Wnt*, and *frizzled* gene candidates across chelicerates, we performed BLAST searches (*P*-value 0.05) against existing genomic and transcriptomic resources and the four new embryonic transcriptomes generated in this study. Hox, Wnt, and Frizzled peptide sequences previously identified in *P. tepidariorum* and *Ixodes scapularis* were reciprocally blasted against the respective NCBI proteomes to confirm their identity and the top hit was selected (Supplementary File S1; Janssen *et al.*^2010^, ^2015^; Schwager *et al.* 2017; Leite *et al.* 2018). Hox protein sequences previously identified in *C. sculpturatus* (Schwager *et al.* 2017) and *P. opilio* (Leite *et al.* 2018) were reciprocally blasted against the respective NCBI proteomes to confirm their identity and the top hit was selected (Supplementary File S1). These sequences, along with the Hox and Fz peptide sequences identified in the *M. martensii* genome, were used as query sequences in our analysis (Di *et al.* 2015; Janssen *et al.* 2015).

BLASTP searches were performed against the NCBI proteomes of *Stegodyphus dumicola*, *C. sculpturatus*, *Tetranychus urticae*, and *Limulus polyphemus*. TBLASTN searches were performed against *C. acosta*, *E. bacillifer*, *P. amentata*, and *M. muscosa* (this study); the transcriptomes of *P. phalangioides* (Turetzek, Torres-Oliva, Kaufholz, Prpic, Posnien, in preparation), *Phoxichilidium femoratum* (Ballesteros *et al.* 2021) and *P. opilio* (PRJNA236471); and the genomes of *Pardosa pseudoannulata* (PRJNA512163), *Acanthoscurria geniculata* (PRJNA222716), and *M. martensii* (CDS sequence file; Cao *et al.* 2013). We then predicted the peptide sequences using the Translate ExPASy online tool (https://web.expasy.org/translate/; default settings). Protein sequence identity was confirmed by reciprocal BLAST against the NCBI database and the construction of maximum-likelihood trees. Where more than one sequence was identified as a potential candidate for a single gene, nucleotide, and protein alignments were inspected to eliminate the possibility that they were isoforms, individual variants, or fragments of the same gene. Only the longest isoforms and gene fragments were selected for phylogenetic analysis (Supplementary File S1). All Hox sequences identified contain a complete or partial homeodomain (Supplementary Files S2 and S3). The TBLASTN search in *P. phalangioides* identified a *Ubx* gene not reported previously by Leite *et al.* (2018). Our BLAST searches did not recover *Cs-ftz-a* (Schwager *et al.* 2017), *Tu-Antp-2* (Grbić *et al.* 2011) or *Is-Wnt8* (Janssen *et al.* 2010) but we included the previously identified sequences in our subsequent phylogenetic analysis. The Trinity transcript accession numbers of all identified sequences, NCBI protein accession and other sequence identifiers used in the subsequent phylogenetic analysis are found in Supplementary File S1.

Due to high levels of fragmentation in the *P. opilio* transcriptome, multiple nonoverlapping fragments were found to align with query *Wnt* sequences. To verify the identity and relationship of these fragments, primers were designed against the 5ʹ-most and 3ʹ-most ends of the aligned series (see Supplementary File S1). Total RNA was extracted using Trizol (Invitrogen) according to the manufacturer’s instructions and cDNA produced using the SuperScriptII first-strand synthesis system (Invitrogen) for RT-PCR using the designed primer pairs. PCR products were sequenced by Macrogen Europe to confirm predicted combinations of fragmented transcripts (see Supplementary File S1).

### Phylogenetic analysis

Hox, Wnt, and Frizzled protein predictions were retrieved from NCBI for the insects *Drosophila melanogaster*, *Bombyx mori*, *Tribolium castaneum*; the crustacean *Daphnia pulex*; and the onychophoran *Euperipatoides kanangrensis* (Supplementary File S1). The Hox, Wnt, and Frizzled peptide sequences for the myriapod *Strigamia maritima* were retrieved from Chipman *et al.* (2014; Supplementary File S1). Alignments were performed in Clustal Omega using default parameters (Larkin *et al.* 2002; Sievers *et al.* 2011), with the exception of the full Hox protein sequences, which were aligned using COBALT (Papadopoulos and Agarwala 2007). Maximum-likelihood trees were generated from whole-sequence alignments to assign sequences to families and study the relationship between candidate duplicates. Phylogenetic analyses were performed in IQ-Tree (v2.0.3, Nguyen *et al.* 2015) using ModelFinder to identify optimal substitution models (JTT + F + R9 for full Hox sequences, LG + G4 for Hox homeodomain sequences, GTR +F + I+ G4 for Hox homeobox sequences, LG + R8 for Wnt, JTT+R8 for Fz; Kalyaanamoorthy *et al.* 2017) and 100,000 bootstrap replicates. Trees were visualized in FigTree v.1.4.4 (http://tree.bio.ed.ac.uk/software/figtree/) and tidied in Adobe Illustrator. Hox sequences were additionally analyzed using RaXML v8 (Stamatakis 2014), using the same substitution models chosen by Iqtree and the automatic bootstopping algorithm (Pattengale *et al.* 2009). Alignments are provided in Supplementary Files S2–S4 and S9–S11.

## Results

### Transcriptome assemblies

To further study the outcomes of WGD in the ancestor of arachnopulmonates, we carried out RNA-Seq on embryos of two further spider species, *P. amentata* and *M. muscosa*, and two species of amblypygids, *C. acosta* and *E. bacillifer*.

RNA-Seq for the four species produced between 222,479,664 and 272,844,971 raw reads, reduced to between 95% and 96.2% after processing. Trinity assembled between 184,142 and 316,021 transcripts in up to 542,344 isoforms (Table 1). Contig N50 ranged from 592 bp in *M. muscosa* to 978 bp in *E. bacillifer*, and from 1461 bp (*M. muscosa*) to 2671 bp (*E. bacillifer*) in the most highly expressed genes (representing 90% of total normalized expression; Table 1).

**Table 1:** Assembly metrics for transcriptomes of *Charinus acosta, Euphrynichus bacillifer, Marpissa muscosa*, and *Pardosa amentata*

Species	Raw reads	Processed reads	#Trinity genes	#Trinity isoforms	Contig N50 (bases)^*a*^	Ex90N50 (bases)^*b*^	#Ex90N50 genes^*c*^	Arachnid BUSCO scores (C[D], F, M)^*d*^	Arthropod BUSCO scores (C[D], F, M)^*e*^
*C. Acosta*	272,844,971	260,853,757	237,678	334,267	896	2,406	31,012	94.9% [12.9%], 1.0%, 4.1%	93.2% [9.5%], 1.5%, 5.3%
*E. bacillifer*	249,938,618	239,034,000	184,142	285,861	978	2,671	22,647	93.8% [7.1%], 1.5%, 4.7%	92.9% [3.5%], 0.7%, 6.4%
*P. amentata*	266,764,548	256,911,378	316,021	542,344	652	1,758	38,423	95.4% [5.3%], 0.9%, 3.7%	92.9% [4.5%], 1.2%, 5.9%
*M. muscosa*	222,479,664	211,848,357	276,943	473,878	592	1,461	46,196	94.7% [6.1%], 1.3%, 4.0%	90.8% [3.9%], 1.9%, 7.3%

a Based on longest isoform per gene.

b Same as contig N50 but based on top most highly expressed genes that represent 90% of the total normalized expression data.

c The number of genes for which Ex90N50 is calculated.

d Ten species, *n* = 2934 BUSCOs; C = Complete [D = Duplicated], F = Fragmented, M = Missing.

e Ninety species, n = 1013 BUSCOs; C = Complete [D = Duplicated], F = Fragmented, M = Missing.

Transcriptomes were found to be between 83.7% (*C. acosta*) and 89.4% (*E. bacillifer*) complete according to BUSCO scores compared to the arthropod database, with between 3.5% and 9.5% duplicated BUSCOs. Compared to the arachnid databases, transcriptomes were 82–90.1% complete for single-copy BUSCOs and contained between 5.3% and 12.9% duplicated BUSCOs (Table 1).

To explore the extent of duplication in these arachnopulmonates, we then surveyed the copy number of *Hox*, *Wnt*, and *frizzled* genes represented in their transcriptomes in comparison to other arachnids. It is important to note that the absence of genes recovered from transcriptomes does not eliminate the possibility that they are present in the genome, as the transcriptomes will only capture genes expressed at the surveyed stages of development. Mixed-stage embryonic samples (all except *E. bacillifer*) may therefore yield more transcripts. See Table 2 for a summary of these details for each species.

**Table 2:** Summary of genetic resources for surveyed arachnopulmonate species

Species	Resource	Stage	No. of individuals
*Acanthoscurria geniculata*	RNA	Mixed	>1
*Centruroides sculpturatus*	DNA	Adult	1
*Charinus acosta*	RNA	Mixed	1
*Euphrynichus bacillifer*	RNA	Single	>1
*Marpissa muscosa*	RNA	Mixed	>1
*Mesobuthus martensii*	DNA	Adult	1
*Parasteatoda tepidariorum*	DNA	Adult	>1
*Pardosa amentata*	RNA	Mixed	>1
*Pardosa pseudoannulata*	DNA	Mixed	>1
*Pholcus phalangioides*	RNA	Mixed	>1
*Stegodyphus dumicola*	DNA	Adult	1

### *Hox* repertoires and their origins

**Figure 1:**
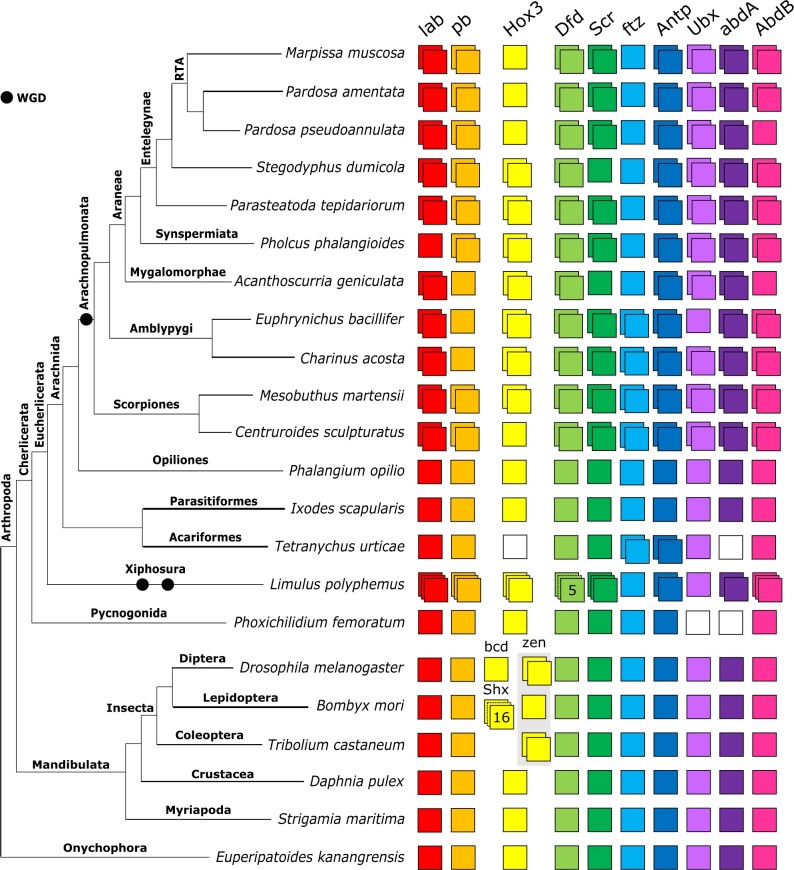
Repertoires of *Hox* genes in arachnids and other selected arthropods. *Hox* genes are represented by colored boxes with duplicated *Hox* genes represented by overlapping boxes and gene loss represented by a white box. Figure includes *Hox* repertoires previously surveyed in the arachnids *P. tepidariorum, Centruroides sculpturatus*, *Mesobuthus martensii*, *Phalangium opilio*, *I. scapularis* (all genomes) and *Pholcus phalangioides* (embryonic transcriptome), the myriapod *Strigamia maritima* and the insects *Drosophila melanogaster*, *Tribolium castaneum*, and *Bombyx mori*. The insect *Hox3* homolog *zen* has undergone independent tandem duplications in *T. castaneum* to yield *zen* and *zen2*; in cyclorrhaphan flies to yield *zen* and *bicoid*; and in the genus *Drosophila* to yield *zen2*. *Bombyx mori* is not representative of all species of ditrysian Lepidoptera, which typically possess four distinct *Hox3* genes termed Special homeobox genes (*ShxA*, *ShxB*, *ShxC*, and *ShxD*) and the canonical *zen* gene.

Spider *Hox* gene repertoires are largely consistent with *P. tepidariorum*, which has two copies of all except *ftz* (Figure 1). There are several exceptions: single copies of *Hox3* in *M. muscosa*, *P. amentata*, and *P. pseudoannulata*, of *Sex combs reduced (Scr)* in *A. geniculata* and *S. dumicola*, of *proboscipedia* (*pb*) in *A. geniculata*, and of *labial* (*lab*) in *P. phalangioides* (Figure 1). Although we found two *AbdB* candidates in *P. pseudoannulata*, one did not contain a homeodomain and was excluded from phylogenetic analyses.

Both amblypygids exhibit extensive duplication of *Hox* genes, with two copies recovered for all except for *pb* in *C. acosta* and *pb* and *Ubx* in *E. bacillifer* (Figure 1).

The scorpions *M. martensii* and *C. sculpturatus* exhibited similar *Hox* repertoires, with two copies recovered for all 10 genes, except for a single copy of *Hox3* in *C. sculpturatus*.

**Figure 2:**
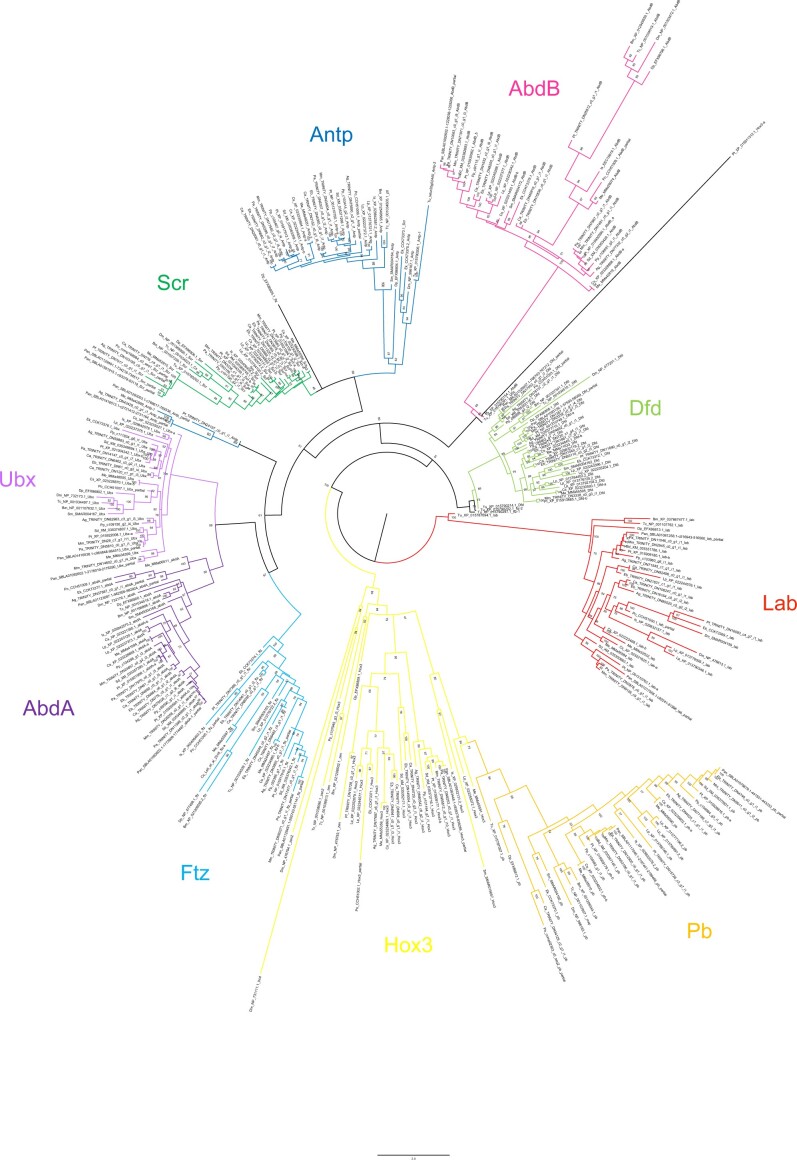
Maximum-likelihood phylogeny of Hox amino acid sequences. The Hox subfamilies are shown as different colors (after Figure 1). Panarthropods included: *Acanthoscurria geniculata* (Ag), *Bombyx mori* (Bm), *Centruroides sculpturatus* (Cs), *Charinus acosta* (Ca), *Drosophila melanogaster* (Dm), *Euperipatoides kanangrensis* (Ek), *Euphrynichus bacillifer* (Eb), *Ixodes scapularis* (Is), *Limulus polyphemus* (Lp), *Marpissa muscosa* (Mm), *Mesobuthus martensii* (Me), *Parasteatoda tepidariorum* (Pt), *Pardosa amentata* (Pa), *Pardosa pseudoannulata* (Pan), *Phalangium opilio* (Po), *Pholcus phalangioides* (Pp), *Phoxichildium femoratum* (Pf), *Stegodyphus dumicola* (Sd), *Strigamia maritima* (Sm), *Tetranychus urticae* (Tu), and *Tribolium castaneum* (Tc). Node labels indicate ultrafast bootstrap support values. See Supplementary File S1 for accession numbers, Supplementary File S2 for full amino acid sequence alignments.

Among the nonarachnopulmonate arachnids, *I. scapularis* and *P*. *opilio* exhibited no duplication of any *Hox* genes, in line with previous genomic surveys (Leite *et al.* 2018; Gainett *et al.* 2021). In the mite *T. urticae*, we did not identify *Hox3* and *abdA* candidates, consistent with Grbić *et al.* (2011) and Ontano *et al.* (2021). The resolution of several sequences from *T. urticae* was variable; for example, previously identified *Tu-ftz-1*, *Tu-ftz-2*, *Tu-AbdB*, and *Tu-Ubx* sequences (Grbić *et al.* 2011) did not resolve correctly in the full Hox tree (Figure 2) but did in the homeodomain and homeobox trees (Supplementary Files S5–S8).

We also surveyed two nonarachnid chelicerates, a pycnogonid *P. femoratum* and the horseshoe crab *L. polyphemus*, the latter of which has undergone multiple rounds of WGD independently of the arachnopulmonates (Kenny *et al.* 2016; Nong *et al.* 2020) we recovered single copies of all *Hox* genes except *Ubx* and *abdA*, which were not found, in *P. femoratum*, consistent with Ballesteros *et al.* (Ontano *et al.* 2021; Figure 1). *Limulus polyphemus* returned multiple copies of all *Hox* genes except *ftz* and *Ubx*, including three copies of *lab*, *pb*, *Hox3*, *Scr*, and *AbdB*, five potential copies of *Dfd*, and two copies of *Antp* and *abdA* (Figure 1).

Full protein sequences produced the best-supported phylogeny: six nodes returned support <50%. Two of these concerned the placement of outgroup (nonchelicerate) sequences and one concerned the deeper relationship of the Hox3 and Pb clades, which is beyond the scope of this study. The remaining three reflect uncertainty in the within-clade placement of chelicerate sequences (Figure 2). An additional 10 nodes returned support of 50–60%, which we consider too weak to justify interpretation. Analyses using protein sequences from homeodomains only contained insufficient phylogenetic information to resolve within-clade relationships, but confirmed gene identity (Supplementary Files S5 and S6). Nucleotide sequences of homeodomains provided more within-clade resolution, but with lower support than full protein sequences (Supplementary Files S7 and S8). Using full protein sequences, we recover Antp as a paraphyletic group, albeit with low support (61%). The homeodomain phylogenies (both protein and nucleotide) confirm the identity of these sequences, indicating substantial sequence divergence outside of this conserved region in several species. The following discussion is based on the phylogeny of the full protein sequences (Figure 2).

Overall, the resolution of duplicate Hox sequences indicates that the majority are likely to be ohnologs. In many cases, duplicates form well-supported clades of orthologs, containing sequences from multiple orders, suggesting a shared origin for duplication. The majority of *Hox* genes are present in duplicate across the arachnopulmonates, strengthening this pattern. Few duplicates form paralogous pairs within species, or paired clades of paralogs within lineages, as would be expected from lineage-specific duplications (although there are exceptions, such as scorpion Lab and Antp, Figure 2).

For both AbdA and AbdB, sequences broadly formed two overall clades; although these were not well supported for AbdB (49–51%), relationships within them are still informative. In both cases, spider sequences formed two distinct and well-supported (>97%) clades that reflect overall phylogenetic relationships in their topology. The amblypygid sequences resolved as two ortholog pairs, one in each of the two overall clades. These are therefore strong candidates for ohnologs. Scorpion AbdB and AbdA were not consistent with this pattern; in both cases, one pair of orthologs resolved together and one pair separately (Figure 2). Although this reflects a low likelihood of a lineage-specific duplication of *abdA* and *AbdB* in scorpions, it does not further clarify possible ohnolog relationships. Spider and scorpion Pb duplicates are candidate ohnologs, resolving in two well-supported (>99%) clades of orthologs whose topologies reflect phylogenetic relationships. Ftz duplicates in scorpions and amblypygids also resolved with orthologs of other arachnopulmonates, but one set of scorpion paralogs was placed with substantial uncertainty (support <60%). Most spider and amblypygid Antp sequences follow the pattern expected of ohnologs, forming two clades of orthologs, but three of the scorpion sequences formed a well-supported clade (91%) while the fourth resolved within a small group of sequences that fall outside the main Antp clade.

The origin of *Ubx*, *Dfd*, *Lab*, *Hox3*, and *Scr* duplicates in arachnopulmonates is not clear from our phylogenetic analysis alone, with neither orthologs nor paralogs resolving together consistently, and topology that is a poor reflection of phylogeny. However, spider sequences broadly formed two clades with other arachnopulmonates, and synteny analysis in both *P. tepidariorum* (Schwager *et al.* 2017) and *Trichonephila antipodiana* (Fan *et al.* 2021) demonstrate clearly that *Hox* cluster duplications therein are the result of WGD. The placement of the amblypygid and scorpion duplicates was more variable. In some cases, these also appear to resolve as expected of ohnologs (*e.g.*, amblypygid Hox3), but in others, their placement could indicate lineage-specific duplication, although usually with low support (*e.g.*, amblypygid Ubx).

### *Wnt* repertoires and their origins

Consistent with *P. tepidariorum* (Janssen *et al.* 2010), we found representatives of 10 *Wnt* subfamilies in all surveyed spiders. The absence of *Wnt9* and *Wnt10* indicates their likely absence in the spider ancestor, while the absence of *Wnt3* is consistent with all other protostomes (Janssen *et al.* 2010; Murat *et al.* 2010; Hogvall *et al.* 2014). Two copies of *Wnt7* were retrieved from *M. muscosa*, *P. amentata*, *A. geniculata*, *P. phalangioides*, *P. tepidariorum*, *P. pseudoannulata*, and *S. dumicola*. All spiders except *A. geniculata* also yielded two copies of *Wnt11*. A second copy of *Wnt4*, which is absent in *P. tepidariorum*, was recovered from *P. amentata*, *M. muscosa*, *A. geniculata*, *S. dumicola*, and *P. pseudoannulata*. We also found duplicates of *Wnt1/wg* in both *A. geniculata* and *P. pseudoannulata*, and a duplicate of *WntA* in *P. pseudoannulata*.

**Figure 3:**
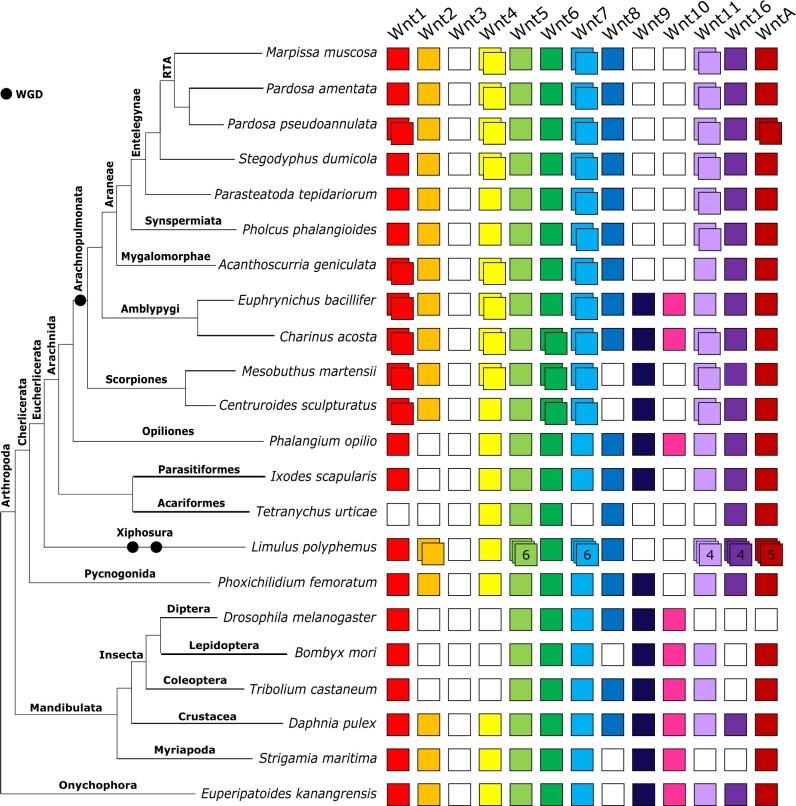
Repertoires of *Wnt* subfamilies in arthropods and an onychophoran. The *Wnt* subfamilies (1–11, 16, and A) are represented by colored boxes with duplicated genes represented by overlapping boxes and putatively lost subfamilies indicated by white boxes. Figure includes *Wnt* repertoires recovered in this study and previously surveyed in the arachnids *Parasteatoda tepidariorum* and *Ixodes scapularis*; the insects *Drosophila melanogaster*, *Tribolium castaneum* and *Bombyx mori*; the crustacean *Daphnia pulex*; the myriapod *Strigamia maritima*; and the onychophoran *Euperipatoides kanangrensis*.

Representation of the *Wnt* subfamilies in the amblypygids is higher than any other chelicerate studied to date, including those with high-quality genome assemblies (Janssen *et al.* 2010; Hogvall *et al.* 2014; Holzem *et al.* 2019). We recovered transcripts from 12 out of 13 subfamilies (missing *Wnt3*) and duplicates of *Wnt1/wg*, *Wnt4*, and *Wnt7* in both species (Figure 3). Additional duplicates of *Wnt6* and *Wnt11* were recovered from *C. acosta* (Figure 3).

Representatives of 10 *Wnt* subfamilies were found in the two scorpion genomes, with both missing *Wnt3*, *Wnt8*, and *Wnt9*. Two copies of *Wnt1/wg*, *Wnt6*, *Wnt7*, and *Wnt11* were retrieved in both species, with an additional copy of *Wnt4* in *M. martensii*.

We did not recover duplicates of *Wnt2*, *Wnt5*, *Wnt8-10*, or *Wnt16* in any arachnopulmonate lineage and found no evidence of *Wnt* gene duplication in the nonarachnopulmonate arachnids (Figure 3). In the harvestman *P. opilio*, we recovered single copies of all except *Wnt2* and *Wnt3*. *Ixodes scapularis* was also missing *Wnt10* but was otherwise similar, whereas we did not recover these or *Wnt1/wg*, *Wnt7*, *Wnt9*, or *Wnt11* in the mite *T. urticae*. In the nonarachnid chelicerates, *P. femoratum* and *L. polyphemus*, we found similar representation of the subfamilies, with all except *Wnt3* and *Wnt10* in *P. femoratum* and all except these and *Wnt9* in *L. polyphemus*. No duplicates were recovered in *P. femoratum*, but large numbers of duplicates were found in *L. polyphemus*. These included six potential copies of *Wnt5* and *Wnt7*, four of *Wnt11* and *Wnt16*, and five of *WntA* (Figure 3).

In our phylogeny using full protein sequences, only two nodes returned support <50%: one uniting Wnt8, Wnt9, Wnt10, and Wnt16 (39%), and one uniting Wnt1/wg, Wnt4, Wnt6, and Wnt11 (36%). These are positioned deep within the tree and concern the interrelationships of the *Wnt* subfamilies, which are beyond the scope of this study.

Most of the duplicate *Wnt* genes appear to be likely ohnologs; confirmation requires synteny analysis, but the relationships between paralogs resolved by phylogenetic analysis generally support duplications originating in the arachnopulmonate ancestor.

**Figure 4:**
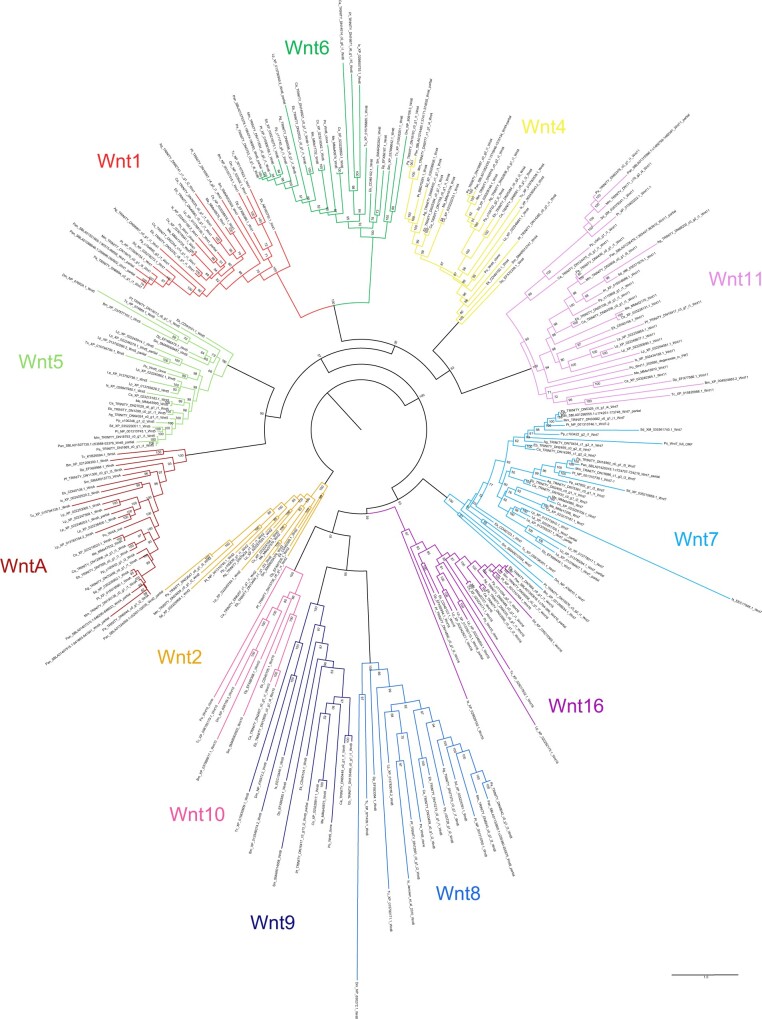
Maximum-likelihood phylogeny of Wnt amino acid sequences. The 12 Wnt subfamilies are shown as different colors (after Figure 3). Panarthropods included: *Acanthoscurria geniculata* (Ag), *Bombyx mori* (Bm), *Centruroides sculpturatus* (Cs), *Charinus acosta* (Ca), *Drosophila melanogaster* (Dm), *Euperipatoides kanangrensis* (Ek), *Euphrynichus bacillifer* (Eb), *Ixodes scapularis* (Is), *Limulus polyphemus* (Lp), *Marpissa muscosa* (Mm), *Mesobuthus martensii* (Me), *Parasteatoda tepidariorum* (Pt), *Pardosa amentata* (Pa), *Pardosa pseudoannulata* (Pan), *Phalangium opilio* (Po), *Pholcus phalangioides* (Pp), *Phoxichildium femoratum* (Pf), *Stegodyphus dumicola* (Sd), *Strigamia maritima* (Sm), *Tetranychus urticae* (Tu), and *Tribolium castaneum* (Tc). Node labels indicate ultrafast bootstrap support values. See Supplementary File S1 for accession numbers, Supplementary File S9 for amino acid sequence alignments, and Supplementary File S10 for nucleotide sequence alignments of *Wnt1/wg* duplicates in *C. acosta, C. sculpturatus*, and *Euphrynichus bacillifer*.

Duplicates of *Wnt7* were previously identified in *P. tepidariorum* (Janssen *et al.* 2010) and are recovered from all surveyed arachnopulmonates. The spider Wnt7 duplicates formed two clades (bootstrap ≥61%) suggesting the retention of ohnologs (Figure 4). The amblypygid Wnt7 ortholog pairs resolve as sisters to the spider clades, but support for these placements is lower (50–60%), and they display slightly higher sequence similarity between paralogs (73–74%) than the spiders (60–72%). The scorpion Wnt7 ortholog pairs formed their own separate clade with strong support (92%; Figure 4), possibly indicating a lineage-specific duplication. Nonetheless, the sequence divergence between the scorpion paralog pairs (68–70%) is similar to that between putative ohnologs in spiders (60–72%).

Duplicates of *Wnt11* previously identified in *P. tepidariorum* (Janssen *et al.* 2010) were recovered from all surveyed spiders except *A. geniculata*, *C. acosta*, and both scorpions. The spider Wnt11 duplicates formed two separate and well-supported clades (≥85%, Figure 4), and each amblypygid Wnt11 orthology group was sister to one of the spider clades (98–99%; Figure 4). Similarity between paralogs in the four new transcriptomes was very low (41–54%). We propose that the spider and amblypygid Wnt11 duplicates are probably retained from the ancestral WGD. The resolution of the scorpion Wnt11 duplicates is less clear; ortholog pairs resolve together with 100% support, but only one resolves as sister to the two clear clades occupied by the spider and amblypygid duplicates (98%; Figure 4). They exhibit similar sequence similarity (49–50%) to the putative ohnologs.

Wnt4 paralogs from *M. muscosa*, *P. amentata*, *A. geniculata*, *P. pseudoannulata*, and *S. dumicola* form two separate and well-supported clades with duplicates from the amblypygids (bootstrap ≥91%). They show substantial sequence divergence within species (53–64% similarity), indicating that they are again likely to represent retained ohnologs following the arachnopulmonate WGD, despite being lost in the lineage to *P. tepidariorum*.

The two sequences of *WntA* recovered from the *P. pseudoannulata* genome are located on the same scaffold and are dissimilar in sequence. Their peptide sequences resolve as sister to one another (Figure 4), indicating a lineage-specific tandem duplication. However, as they are partial sequences with a short overlapping region, this requires confirmation.

We identified two copies of *Wnt1/wg* in the transcriptomes of both amblypygids and *A. geniculata*, and in the genomes of *P. pseudoannulata*, *C. sculpturatus*, and *M. martensii*. Sequence similarity between paralogs was low (55–73%) compared to similarity between orthologs at the order level (*e.g.*, 91% between *M. muscosa* and *P. amentata*), and comparable to orthologs at the class level (*e.g.*, 61% between *P. tepidariorum* and *I. scapularis*). Although synteny analysis is required for conclusive confirmation, our phylogeny indicates that the amblypygid, scorpion, and *A. geniculata* duplicates are likely to be ohnologs retained from the arachnopulmonate WGD, as they form separate clades (≥70%, Figure 4). The putative duplicates in *P. pseudoannulata*, in contrast, resolve as sister to one another. These two sequences have (short) nonidentical overlapping regions, but, again, they are partial; therefore, it is equivocal whether they represent a genuine, lineage-specific duplication.

### *Frizzled* repertoires and their origins

**Figure 5:**
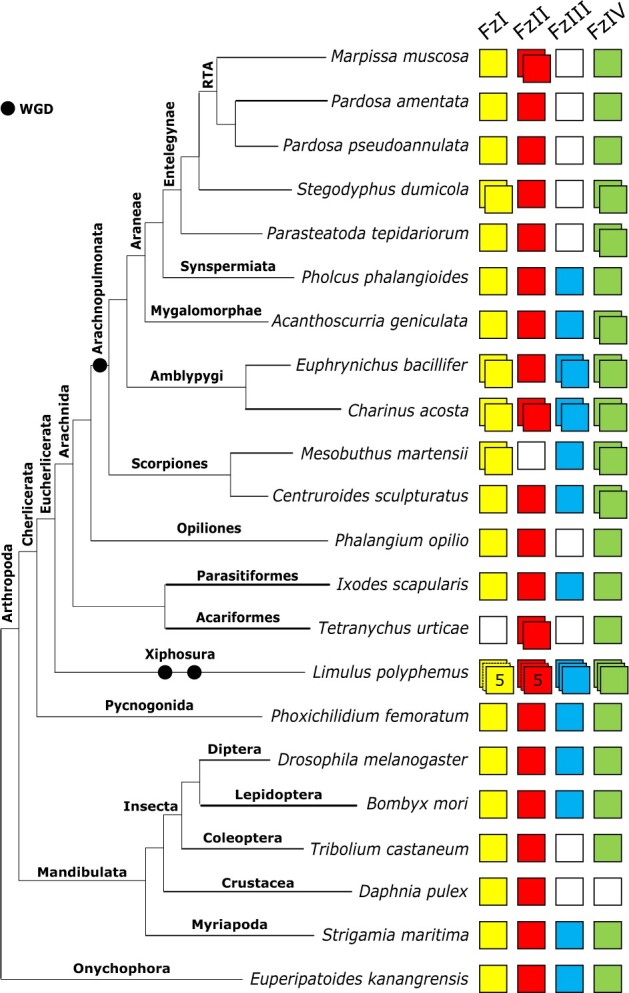
Repertoire of *frizzled* genes in arachnids and other selected arthropods. The four *frizzled* subfamilies (FzI, FzII, FzIII, and FzIV) are represented by colored boxes, with duplicated genes represented by overlapping boxes and gene loss represented by a white box. Figure includes *frizzled* repertoires previously surveyed in the arachnids *Parasteatoda tepidariorum*, *Mesobuthus martensii*, *Ixodes scapularis*, and *Pholcus phalangioides*; the myriapod *Strigamia maritima*; the insects *Drosophila melanogaster* and *Tribolium castaneum*; and the onychophoran *Euperipatoides kanangrensis*.

All surveyed spiders possess at least one ortholog of FzI and FzII. A second ortholog of FzI was recovered from the genome of *S. dumicola* and a second ortholog of FzII from the transcriptome of *M. muscosa* (Figure 5). FzIII was absent from the transcriptomes of *M. muscosa* and *P. amentata*, and the genomes of *S. dumicola* and *P. pseudoannulata*, consistent with *P. tepidariorum* (Janssen *et al.* 2015). However, single FzIII orthologs were recovered from *A. geniculata* and previously identified in *P. phalangioides* (Janssen *et al.* 2015). Consistent with *P. tepidariorum* (Janssen *et al.* 2015), we identified two FzIV sequences in *S. dumicola* and *A. geniculata*, but only one in *M. muscosa*, *P. amentata*, and *P. pseudoannulata* and only one was previously recovered from *P. phalangioides* (Janssen *et al.* 2015).

The two amblypygid species have a large repertoire of *frizzled* genes compared to other arachnids, with duplicates of FzI, FIII, and FzIV orthologs in both species, as well as a duplication of FzII in *C. acosta* (Figure 5).

The scorpions *M. martensii* (Janssen *et al.* 2015) and *C. sculpturatus* possess single FzI and FzIII orthologs and a duplication of FzIV. We also recovered a single FzII ortholog in *C. sculpturatus*, and an FzI duplicate in *M. martensii*.

Among the nonarachnopulmonate arachnids, *I. scapularis* possesses a single copy of all four subfamilies, but FzIII was absent in *P. opilio* and *T. urticae*. A FzII duplicate was recovered from *T. urticae. Phoxichilidium femoratum* and *L. polyphemus* possess all four subfamilies, and while no duplicates were recovered in *P. femoratum*, five copies of FzI and FzII and three copies of FzIII and FzIV were recovered from *L. polyphemus* (Figure 5).

**Figure 6:**
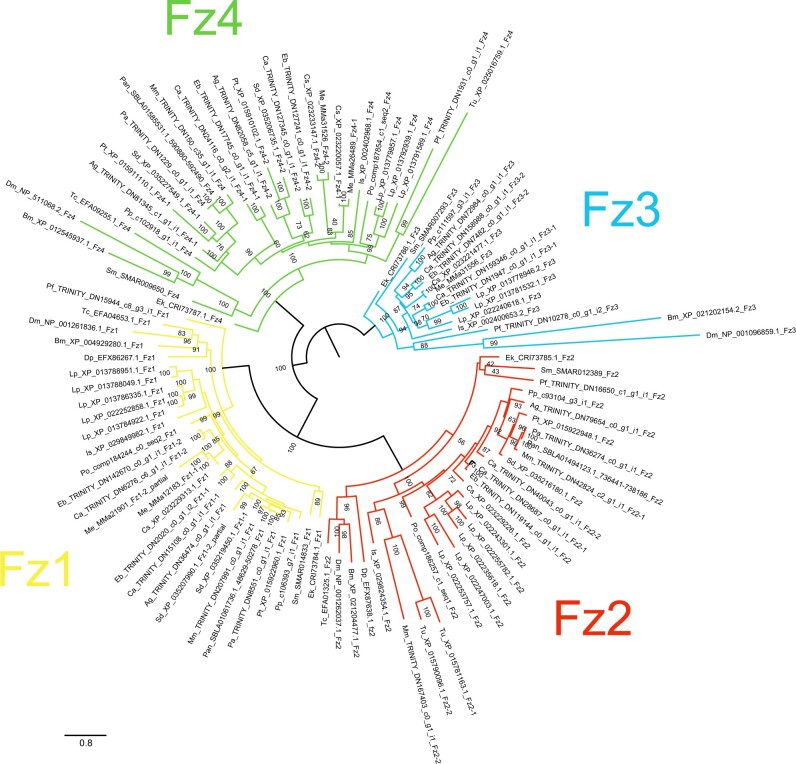
Maximum-likelihood phylogeny of Frizzled proteins. The Frizzled subfamilies are shown as different colors (after Figure 5). Panarthropods included: *Acanthoscurria geniculata* (Ag), *Bombyx mori* (Bm), *Centruroides sculpturatus* (Cs), *Charinus acosta* (Ca), *Drosophila melanogaster* (Dm), *Euperipatoides kanangrensis* (Ek), *Euphrynichus bacillifer* (Eb), *Ixodes scapularis* (Is), *Limulus polyphemus* (Lp), *Marpissa muscosa* (Mm), *Mesobuthus martensii* (Me), *Parasteatoda tepidariorum* (Pt), *Pardosa amentata* (Pa), *Pardosa pseudoannulata* (Pan), *Phalangium opilio* (Po), *Pholcus phalangioides* (Pp), *Phoxichildium femoratum* (Pf), *Stegodyphus dumicola* (Sd), *Strigamia maritima* (Sm), *Tetranychus urticae* (Tu), and *Tribolium castaneum* (Tc). Node labels indicate ultrafast bootstrap support values. See Supplementary File S1 for accession numbers and Supplementary File S11 for alignments.

*Frizzled* paralogs appear to stem from both WGD events and lineage-specific duplications. FzI duplicates were recovered from both amblypygid transcriptomes*, M. martensii*, and *S. dumicola.* The Sd-Fz1 and Me-Fz1 paralog pairs exhibit high sequence similarity (>73%) and resolved as sisters with 100% bootstrap support, indicating that these are likely the results of lineage-specific duplications. Conversely, the amblypygid Fz1-1 and Fz1-2 ortholog pairs form separate clades with sequences from spiders and scorpions, respectively (Figure 6). We therefore interpret these duplicates as ohnologs.

Duplicates of FzII were recovered from *M. muscosa* and *C. acosta*. The Mm-Fz2 paralogs form a well-supported clade (79%; Figure 6), indicating that this is the result of a lineage-specific duplication followed by sequence divergence in Mm-Fz2-2 (53% sequence similarity). The origin of the FzII duplication in *C. acosta* is not clear. Ca-Fz2-1 resolves as a sister group to the spider sequences (99%; Figure 6) and Ca-Fz2-2 forms a clade with Eb-Fz2, which in turn resolves as sister to the Ca-Fz2-1 and spider sequences (93%; Figure 6). This topology could support an ohnolog relationship between Ca-Fz2-1 and Ca-Fz2-2 but cannot be confirmed. Sequence similarity between the *C. acosta* paralogs is relatively high (82%), perhaps higher than expected from ohnologs.

The origin of the amblypygid FzIII duplicates is also unclear. One ortholog pair forms a clade with *L. polyphemus* sequences (66%; Figure 6) and the other forms a clade with the spiders and scorpions (≥70%; Figure 6). This suggests an origin in WGD, but support for their placement is not strong (66%, Figure 6). Paralogous pairs demonstrate middling sequence similarity (65–66%).

Both amblypygids and scorpions, and the spiders *P. tepidariorum*, *S. dumicola*, and *A. geniculata*, possess FzIV duplicates. The spider sequences formed two separate and well-supported clades (100%; Figure 6). The amblypygid ortholog pairs resolved as sister to the two spider clades (98%; Figure 6). The scorpion ortholog pairs, however, formed a clade together (76% support; Figure 6) which is sister to all the spider and amblypygid sequences (82%; Figure 6). All paralogous pairs exhibited substantial sequence divergence (similarity 44–58%). We propose that the spider and amblypygid FzIV duplicates are retained from the ancestral WGD; however, once again, the origin of the scorpion FzIV duplicates is less clear, and may reflect a lineage-specific duplication.

## Discussion

The contribution of WGD to expanding the developmental toolkit is evident across the three gene families surveyed here, but it appears that retention patterns of putative ohnologs vary substantially between them. We also see distinct phylogenetic patterns beginning to emerge in gene repertoires, with improved sampling within lineages enabling us to distinguish between possible signal and likely noise. Of course, transcriptomic data come with necessary caveats regarding gene expression and capture, and absences from transcriptomes should be regarded with caution. However, where patterns are consistent across multiple species, or better, between transcriptomic and genomic resources, we can be more confident about their authenticity. For example, comparisons of the transcriptome of *P. amentata* and the genome of *P. pseudoannulata* show identical *Hox* and *frizzled* repertoires, indicating good gene capture in the former. The apparent duplicates of *Wnt1/wg* and *WntA* in *P. pseudoannulata* that were not recovered in *P. amentata* are of uncertain status (partial sequences, phylogenetic position) and were absent from all other spider genomes. Overall, levels of gene duplication detected using BUSCO were in line with other arachnopulmonates [*e.g.*, 3.3% in *Argiope bruennichi*, Sheffer *et al.* (2021); 5.6% in *T. antipodiana*, Fan *et al.* (2021); 11% in *Araneus ventricosus*, Kono *et al.* (2019)] but lower than in horseshoe crabs [*e.g.*, 16.7% in *Carcinoscorpius rotundicauda*, Shingate *et al.* (2020)].

### *Hox* duplicates are broadly retained

The widespread retention of duplicate *Hox* genes is consistent among the arachnopulmonate orders studied to date, and specific repertoires appear to be fairly conserved at the order level (Schwager *et al.* 2007; Cao *et al.* 2013; Di *et al.* 2015; Schwager *et al.* 2017; Leite *et al.* 2018; Ontano *et al.* 2021). Given that this is the level at which overall body plans are conserved, this is perhaps not surprising. Thanks to the relatively conserved expression patterns of *Hox* genes along the antero-posterior axis of chelicerates, we can begin to speculate about the possible macroevolutionary implications of duplication and loss.

The absence of a second copy of *pb* in both *C. acosta* and *E. bacillifer*, which are distantly related within Amblypygi, suggests a loss in the common ancestor of amblypygids. Gainett and Sharma (2020) also recovered a single copy of *pb* in *P. marginemaculatus*, but two copies in *Charinus israelensis*. However, one of these sequences (32 aa) had an incomplete homeodomain that was identical to the other copy; therefore, we are hesitant about its status as a duplicate. Embryos of *C*. *acosta* were collected at multiple stages of development, supporting the hypothesis that this is a genuine loss, rather than absence of expression at a particular developmental stage. In spiders, both *pb* ohnologs are expressed in the pedipalp and leg-bearing segments, separated temporally (Schwager *et al.* 2017). Given the highly derived nature of the raptorial pedipalps and the antenniform first pair of walking legs in amblypygids, it is perhaps surprising that this duplicate was not retained. However, this might indicate that other Hox genes expressed in the anterior prosomal segments (*e.g.*, *lab*, *Hox3*, or *Dfd*) may contribute to these morphological innovations. Recent work by Gainett and Sharma (2020) examining the specification of the antenniform legs found little difference in *Distal-less*, *dachshund*, or *homothorax* expression between that and posterior leg pairs, indicating that these are not likely to be responsible. A good candidate for future study might be *lab*: a single ortholog is expressed in both the pedipalps and the first walking leg in the harvestman *P. opilio* (Sharma *et al.* 2012), and expression patterns and experimental manipulation provide evidence for functional divergence between the two *lab* ohnologs, which are expressed in the pedipalps and first walking legs, in *P. tepidariorum* (Pechmann *et al.* 2015; Schomburg *et al.* 2020).

While the *ftz* duplicate has been retained in amblypygids and scorpions, it seems likely that having a single copy of *ftz* is common across all spiders (Figures 1 and 2), consistent with the loss of a duplicate in their common ancestor. A recently published high-quality genome from *T. antipodiana* also reported a single copy of *ftz* (Fan *et al.* 2021), as did another mygalomorph transcriptome, *A. hentzi* (Ontano *et al.* 2021). In both *P. opilio* (Sharma *et al.* 2012) and *P. tepidariorum* (Schwager *et al.* 2017), a single copy of *ftz* is expressed in leg pairs 2–4. Any subfunctionalization or neofunctionalization that could be evident in scorpion and amblypygid *ftz* had therefore presumably not taken place at the point of their divergence from spiders. Expression patterns of the two paralogs in these groups would be of interest for comparison with both harvestmen and spiders.

The absence of *Hox3* duplicates in *M. muscosa*, *P. pseudoannulata*, and *P. amentata* may indicate a lineage-specific loss in the RTA clade, which unites salticids, lycosids, and their allies. Indeed, only one copy of *Hox3* was previously recovered in *Cupiennius salei*, which also belongs to the RTA clade (Schwager *et al.* 2007). Although the absence of a second copy in the embryonic transcriptomes could be attributed to failure to capture expression, both *C. salei* and *P. pseudoannulata* yielded single copies from DNA, strengthening the case for a genuine loss. In *P. tepidariorum*, Hox3-A and Hox3-B exhibit dramatic protein sequence divergence (20.6% similarity). Both copies are expressed in the embryo, with their expression overlapping spatially and temporally but not identical, indicating some functional divergence. *Hox3-A* expression was reported to be very weak (Schwager *et al.* 2017), and the Hox-3A homeodomain demonstrates much higher divergence from other arachnid Hox3 sequences than does Hox3-B (Supplementary File S3); together, these could reflect diminished functionality. In the other spiders for which we recovered *Hox3* duplicates, paralogs also exhibited very low sequence similarities (24.7–30.6%), and these duplicates had very low similarity to each other (17.2–30.1%, compared to 33–58.2% between those resolving with other spider Hox3 sequences). In all cases, one resolved within a well-supported clade of spider Hox3 and the other was placed haphazardly. We speculate that the *Hox3* duplicate is highly divergent or degenerate in spiders, leading to eventual pseudogenization in the RTA clade.

Other apparent losses of *Hox* gene duplicates are restricted to transcriptomes of individual species, with the exception of *Scr* in *S. dumicola*, and could reflect failure to capture additional sequences or indicate lineage-specific loss. It would be premature to conclude that they are genuinely absent from the genome. For instance, the apparent absence of a *Ubx* duplicate in *E. bacillifer*. In *P. tepidariorum*, the two *Ubx* ohnologs are expressed most strongly in close succession, at stages 8.1 and 8.2 (Schwager *et al.* 2017), but this may not reflect their relative expression patterns in amblypygids, and therefore one may not have been captured in this single-stage transcriptome.

### Limited retention of *Wnt* duplicates

In contrast to the widespread retention of *Hox* duplicates, these new data indicate that the retention of duplicate *Wnt* genes is less common and restricted to certain subfamilies. Apparent ohnologs of *Wnt4*, *Wnt7*, and *Wnt11* are retained in the majority of arachnopulmonates, for example, but *Wnt2*, *Wnt5*, *Wnt8-10*, *Wnt16*, and *WntA* are only represented by single copies. These patterns could reflect early losses of duplicates, and/or differential effects of higher copy numbers across *Wnt* subfamilies. The fact that retention patterns appear to differ between the arachnopulmonate and horseshoe crab independent WGD events lends support to the former hypothesis.

Our understanding of specific Wnt functions among arthropods is more limited than that of *Hox* genes, but *Wnt* expression patterns in *P. tepidariorum* are available for tentative comparison. For example, previous attempts to characterize the expression patterns of *Wnt11* paralogs in *P. tepidariorum* only detected expression of *Wnt11-2* (Janssen *et al.* 2010). Given the retention of *Wnt11-1* in both spiders and scorpions, and the considerable divergence between paralogous sequences, *Wnt11* could be a good candidate for sub- or neofunctionalization, but the role of *Wnt11-1* remains unknown. Conversely, the presence of two apparent *Wnt4* ohnologs in spiders and amblypygids (Figures 3 and 4) contrasts with the retention of only one *Wnt4* in *P. tepidariorum*. The role of the additional copy, particularly in spiders, will be of future interest and expression patterns of *Wnt4-2* in these groups will help to clarify this. Although it is possible that the second copy is redundant or in the process of pseudogenization, the fact that both ohnologs are retained in two large clades, with detectable levels of expression during development, suggests that this is not the case. Thus, its absence in *P. tepidariorum* unexpectedly appears to be the exception.

The discovery of duplicate *Wnt1/wg* in scorpions, amblypygids, and mygalomorphs is particularly exciting: duplicates of this Wnt gene have not yet been detected in any other metazoans, even following multiple rounds of WGD in vertebrates, teleosts (see https://web.stanford.edu/group/nusselab/cgi-bin/wnt/vertebrate), or horseshoe crabs. This requires critical interpretation. We can eliminate the possibility of individual variation in *C. acosta*, as embryos are produced by parthenogenesis and are therefore clones (de Armas 2005), and in the two scorpions, as the sequences were recovered from a single individual’s genome (Supplementary File S1). Levels of sequence divergence exceed those seen between putative tandem duplications, in both nucleotide and amino acid sequences. Wnt1/wg performs a wide variety of roles in arthropods, including in segment polarization and in appendage and nervous system development (Murat *et al.* 2010) and has an accordingly complex expression pattern in *P. tepidariorum*, appearing in the L1 and L2 segments, limb buds, and dorsal O2 and O3 segments (Janssen *et al.* 2010). In theory, therefore, there is ample potential for subfunctionalization. Gene expression and functional studies of *Wnt1/wg* duplicates in arachnopulmonates will no doubt prove extremely interesting in the future.

The presence of *Wnt10* in both amblypygids and *P. opilio* is also intriguing because it is absent from all other chelicerates surveyed so far. This could indicate multiple losses of *Wnt10* in all other arachnid lineages, the co-option of another gene in amblypygids and harvestmen, or the recovery of a lost *Wnt10*; however, the possible routes for co-option are unclear and pseudogene recovery has only been suggested in a handful of cases (Trabesinger-Ruef *et al.* 1996; *e.g.*, Balakirev and Ayala 2003). It is notable that we did not recover *Wnt10* orthologs in *L. polyphemus* or *P. femoratum*, nonarachnid chelicerates. Studies of other available horseshoe crab genomes (Shingate *et al.* 2020; Nong *et al.* 2020) will shed further light on whether *Wnt10* is truly absent.

### *frizzled* repertoires vary substantially

Previous studies of spiders, scorpions, and ticks indicated that *frizzled* repertoires in these groups are restricted to three or four genes, often with incomplete representation of the four subfamilies (Janssen *et al.* 2015). The spiders *M. muscosa*, *S. dumicola*, *P. pseudoannulata*, and *P. amentata* are consistent with this pattern, albeit with a unique duplication of FzII in the jumping spider. The presence of FzIII duplicates in *P. phalangioides* and *A. geniculata* indicates that, while entelegynes may universally lack *fz3*, it was likely present in the ancestor of all spiders. In contrast, all four *frizzled* subfamilies were recovered in both amblypygid species, with three present in duplicate in *E. bacillifer* and four in *C. acosta*. Based on our data, it appears that the *frizzled* repertoire of amblypygids is around twice the size of all other arachnids and may have followed a very different evolutionary trajectory to spiders and scorpions following WGD. The expanded repertoire of *frizzled* genes in amblypygids is intriguing since they have the largest *Wnt* repertoires, via both duplication and representation of the subfamilies (Wu and Nusse 2002). However, although *frizzled* genes encode key receptors for Wnt ligands, they have other Wnt-independent functions, so the expansion of the *frizzled* repertoire could be equally related to the evolution of alternative signaling roles (Janssen *et al.* 2015; Yu *et al.* 2020).

### Arachnopulmonate genome evolution in the wake of WGD

Our new analyses provide a thorough survey of *Hox*, *Wnt*, and *frizzled* genes in arachnids, and substantially improve the density and breadth of taxonomic sampling for these key developmental genes in Arachnopulmonata. We find evidence of consistent evolutionary trajectories in *Hox* and *Wnt* gene repertoires across three of the six arachnopulmonate orders, with interorder variation in the retention of specific paralogs. We have also identified intraorder variation at the level of major clades in spiders, which could help us better understand their morphological evolution. In new data for a third arachnopulmonate lineage, the amblypygids, we find additional evidence supporting an ancestral WGD and are better able to reconstruct the chronology of gene duplications and losses in spiders and scorpions. These transcriptomic resources are among the first available for amblypygids and will aid future investigations of this fascinating group.

By improving taxonomic coverage within the spider lineage, we are better able to polarize some loss/duplication events and identify potential new trends within the spiders, particularly illustrating separations between synspermiatan and entelegyne spiders, and between the derived RTA clade and other spiders. Despite being unable to ultimately conclude that some missing transcripts reflect genuine genomic losses, it appears that the evolution of these developmental genes in spiders is more complicated than we thought. It may be that these gene repertoires are genuinely more variable within spiders than they are in amblypygids or scorpions; spiders are by far the most taxonomically diverse arachnopulmonate order, and the apparent diversity of repertoires may simply reflect this. Conversely, the higher apparent intraorder diversity of gene repertoires may be an artifact of increased sampling in spiders (up to four or five species for specific gene families) compared to the one or two available resources for scorpions and amblypygids; we may detect more diversity within these groups with increased sampling. Nonetheless, we see two notable trends within spiders, outlined below.

First, we see several characters that appear to unite the RTA clade, which contains almost half of all extant spider species (World Spider Catalog 2019), having diversified rapidly following its divergence from the orb weavers (Garrison *et al.* 2016; Fernández *et al.* 2018; Shao and Li 2018). *Marpissa muscosa*, *P. amentata*, and *P. pseudoannulata* all exhibit the apparent loss of *Hox3* and *fz4* paralogs and the retention of a *Wnt4* duplicate. The identification of genetic trends potentially uniting this group is exciting, even if the macroevolutionary implications are unclear: as described above, the possible functions of a *Wnt4* paralog are elusive. Members of the RTA clade are very derived compared to other araneomorph spiders, both morphologically (*e.g.*, male pedipalp morphology and sophisticated eyes) and ecologically (most are wandering hunters), and their rapid diversification would align with clade-specific genetic divergence (Garrison *et al.* 2016; Fernández *et al.* 2018; Shao and Li 2018).

Second, although data are only available for single representatives of the plesiomorphic clades Synspermiata (*P. phalangioides*) and Mygalomorphae (*A. geniculata*), these hint at lineage-specific losses of *Hox* paralogs and recover the only examples of FzIII found in spiders so far. The presence of FzIII is consistent with other arachnopulmonate groups and suggests that it was present in the spider ancestor and only lost in the more derived entelegyne lineages. If selected *Hox* duplicates are indeed absent from the genomes of these two species, this could represent an interesting divergence between the three major groups of spiders. Though these genes are unlikely to be directly responsible, the divergence might provide a starting point for understanding the important morphological differences between mygalomorphs, synspermiatans, and entelegynes. However, genomic information for additional taxa in both groups is required to verify these potential losses.

The amblypygids emerge as a key group of interest for studying the impacts of WGD owing to their high levels of ohnolog retention. Our transcriptomes, from representatives of two major clades, provide further evidence supporting a WGD in the ancestor of arachnopulmonates and demonstrate widespread retention of ohnologs in three major families of developmental genes (consistent with the retention of many duplicated regulators of eye development in other species, Gainett *et al.* 2020). In all three gene families we studied, repertoires were largest in the amblypygid species. This was particularly the case in *C. acosta*, which belongs to the less speciose and more plesiomorphic infraorder Charinidae within living Amblypygi. Although this study represents just two amblypygid species and three gene families, this appears to contradict widespread predictions of diversification with the duplication of important developmental genes such as *Hox* (*e.g.*, Van De Peer *et al.* 2009). Of particular interest are the amblypygid *Wnt* gene repertoires. We have identified from their transcriptomes, and from the published genome of *C. sculpturatus*, the first reported duplicates of *Wnt1/wg* in any animal, as well as the first reported *Wnt10* in any arachnid. Future functional studies of these genes and their expression during development will be critical to understanding the evolutionary impacts of these unusual components of amblypygid gene repertoires. Amblypygids also represent a potential model group for studying the evolution of arthropod body plans, owing to the unusual and derived morphology of the pedipalps and especially the first walking legs. Thanks to a substantial existing body of work on anterior-posterior patterning, segmentation, and appendage development in spiders and other arachnids, we may have a chance to crack the genetic underpinnings of these dramatic evolutionary innovations (Pechmann *et al.* 2009; Sharma *et al.*^2012^, ^2014^a; Turetzek *et al.* 2016, 2017; Schwager *et al.* 2017; Schomburg *et al.* 2020; Baudouin-Gonzalez *et al.* 2021).

Finally, our analysis of existing genomic data for *C. sculpturatus* and *M. martensii* has recovered several *Wnt* and *frizzled* gene duplications, similar to spiders and amblypygids. However, in contrast to those groups, our phylogenies have sometimes supported within-lineage duplication in scorpions, as opposed to the retention of ohnologs following WGD, even when these are observed in spiders and amblypygids. This was the case for *AbdB*, *Wnt1/wg*, *Wnt6*, *Wnt7*, and FzIV (Figures 2, 4, and 6). However, levels of sequence similarity in these cases were comparable for *C. sculpturatus* paralogs and amblypygid and spider ohnologs, when we might expect within-lineage duplicates to show higher similarity. The resolution of the paralogous sequences in our phylogenetic analyses could be confounded by the early-branching position of scorpions within Arachnopulmonata, which means paralogs would be expected to appear toward the bottom of ortholog clades and are more vulnerable to movement. Nonetheless, this pattern emerged multiple times in our analyses and may be of future interest.

Both arachnopulmonates and horseshoe crabs have been subject to WGD. Comparison between these independent events is a useful tool in studying WGD, spanning smaller phylogenetic distances than arachnopulmonate-vertebrate comparisons. From the three gene families surveyed here, both patterns and inconsistencies emerge. As in arachnopulmonates, *Hox* gene duplicates appear to be overwhelmingly retained, even in triplet or quadruplet in some cases, in *L. polyphemus*. *Ftz* is an interesting exception that aligns with the absence of duplicates in spiders, but not scorpions or amblypygids. However, the apparent loss of all *Ubx* and two *abdA* duplicates stands at odds with the arachnopulmonates, wherein these are largely retained. As seen in arachnopulmonates, only select *Wnt* genes are retained in duplicate in *L. polyphemus*. However, it is not always the same *Wnts*: for example, whereas *Wnt4* duplicates are common in arachnopulmonates, they are completely absent in *L. polyphemus*, and vice versa for *Wnt5*. There are, however, some *Wnts* that are commonly or even universally present in single copies following both independent WGD events, potentially indicating low potential for sub- or neofunctionalization, such as *Wnt8*, *Wnt6*, and *Wnt1*/*wg*.

Overall, our new data provide further evidence of an ancestral arachnopulmonate WGD, identify evolutionary patterns within gene families following WGD, reveal new diversity in spider gene repertoires, better contextualize existing data from spiders and scorpions, and broaden the phylogenetic scope of available data for future researchers. However, other arachnid groups, both with and without ancestral WGD, require further study. Recent work on two pseudoscorpions recovered duplicates of most *Hox* genes (Ontano *et al.* 2021). This not only further supports arachnopulmonate WGD but substantially improves our understanding of pseudoscorpion placement within arachnids, which has been historically problematic (*e.g.*, Ballesteros *et al.* 2019; Ballesteros and Sharma 2019; Arribas *et al.* 2020). The sequencing of a pseudoscorpion genome provides the tantalizing chance to add a fourth lineage to future studies of WGD and its impacts (Ontano *et al.* 2021). The remaining arachnopulmonate orders, thelyphonids (vinegaroons or whip scorpions) and schizomids, form a clade with amblypygids (Pedipalpi) and should also have been subject to the arachnopulmonate WGD. Future work on these groups will shed light on the unusual patterns of gene retention we find in both major clades of amblypygids. Nonarachnopulmonate arachnids are invaluable for contextualizing the changes that occur following WGD, both in terms of gene repertoires and of gene function. Genomic resources and gene expression pattern studies are vital for this, and the harvestman *P. opilio* has emerged as the clear model species (Sharma *et al.* 2012; Gainett *et al.* 2021). The ability to compare rates of sequence divergence, within-lineage gene duplication, and, eventually, functional properties of developmental genes in these groups will provide critical comparative data for arachnopulmonates.

## Data availability

Alignments and gene accession numbers are included as supplementary information files. Reads and assembled transcriptomes are available on SRA and TSA, respectively, under BioProject PRJNA707377. Supplemental material is available at figshare: https://doi.org/10.25387/g3.14456289.
